# 

**Published:** 1880-10

**Authors:** 


*CONTENTS.*

PAGE
THE LITTLE COURTESIES OF LIFE 173
BEQUESTS TO CHILDREN........ 175
SLEEP........................ 175
IMPUDENCE VS. CLIMATE....... 176
INFANTS' FOOD................ 177
CELIBACY..................... 177
THE THREE P'S................ 179
COFFEE DRINKING.............. 180
PURE AIR..................... 181
CROUP........................ 182
NIGHT AIR.................... 182

PAGE
STRIKING SENTIMENTS......... 185
HUMAN HOPES................. 187
LITERATURE OF THE TIMES..... 188
THINK WELL RATHER THAN EVIL 189
SEARCH FOR WIVES............ 190
LEAD POISON................. 191
SECOND CHILDHOOD............ 192
PARENTAL CORRECTIONS........ 192
HEARTY SUPPERS.............. 199
TRIALS OF LIFE.............. 202

